# The mitochondrial genome of the pentastome parasite *Raillietiella orientalis* Hett, 1915 (Raillietiellida; Raillietiellidae) with notes on its phylogenetic position

**DOI:** 10.1080/23802359.2023.2224461

**Published:** 2023-07-18

**Authors:** Jenna N. Palmisano, Terence M. Farrell, Taryn M. Gustafson, Robert R. Fitak

**Affiliations:** a Department of Biology, University of Central Florida, Orlando, FL, USA; b Department of Biology, Stetson University, DeLand, FL, USA

**Keywords:** Mitogenome, invasive, crustacean

## Abstract

In this study we sequenced and annotated the complete mitochondrial genome of the invasive reptile parasite *Raillietiella orientalis* using Illumina DNA sequencing. The length of the mitogenome was 15,320 bp and had a GC content of 33.1%. The mitogenome contained 13 protein-coding genes, two ribosomal RNA genes, and 22 tRNA genes, the order of which was diagnostic of Pancrustacean mitogenomes. A phylogenetic tree constructed from the 13 protein-coding genes of *R. orientalis* and 26 other Pancrustacean mitogenomes supported the placement of *R. orientalis* as part of the monophyletic subclass Pentastomida within the Maxillopoda and sister to the subclass Branchiura.

Pentastomes are endoparasitic crustaceans that largely parasitize the lungs of carnivorous reptiles (Pare 2008). The pentastome *Raillietiella orientalis* (Hett 1915) (Figure 1) is native to southeast Asia where it infects a diverse assemblage of herpetofauna including snakes and large lizards as definitive hosts, anurans and small lizards as secondary intermediate hosts, and invertebrates as primary intermediate hosts (Walden et al. 2020; Fieldsend et al. 2021; Palmisano et al. 2022). The abundant and diverse set of intermediate and definitive hosts infected by *R. orientalis* facilitates its establishment and spread in novel systems. There are currently two known invasion fronts of *R. orientalis* – Australia and North America – where infections in native snake populations are severe and often fatal (Kelehear et al. 2014; Farrell et al. 2019; Bogan et al. 2022). The origins of *R. orientalis* invasions are poorly understood, however, introduced populations of Burmese pythons in Florida may have been the initial source of parasite spillover in North America (Miller et al. 2018).

**Figure 1.**
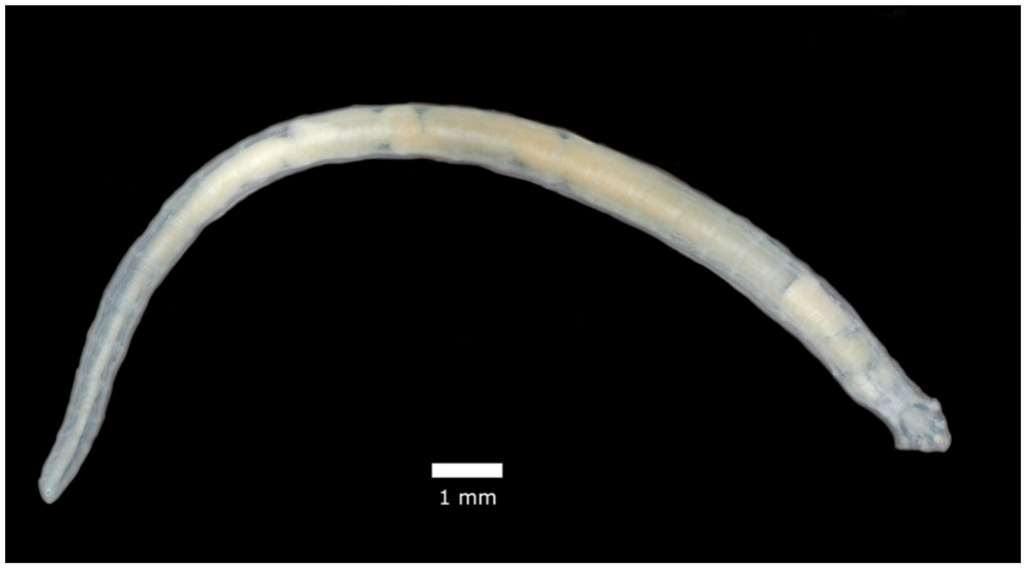
Reference image of an adult female *Raillietiella orientalis*. This image is original work provided by the authors.

The taxonomy and systematics of the Pentastomida have long been disputed. Originally, this monophyletic clade was classified as its own phylum, but was subsequently repositioned to a subclass of Crustaceans (Lavrov et al. 2004). Over 130 species of pentastomids are recognized and distributed amongst two orders: Cephalobaenida and Porocephalida. Two genera in the Porocephalida, *Armillifer* and *Linguatula*, occur as zoonoses and have published mitogenomes (Lavrov et al. 2004; Naude et al. 2018). *Raillietiella orientalis* belongs to the Cephalobaenida for which no genomic resources are available. To help resolve their phylogenetic relations with other pentastomids, and to provide information that might be useful to researchers studying the conservation problems this invasive species poses, we present the mitochondrial genome of *R. orientalis*.

A deceased female southern black racer (*Coluber constrictor*) was discovered on the road of a residential neighborhood in Seminole County, Florida, USA (28.6349263° N, 81.2773628° W). The snake had *R. orientalis* adults exiting out of her glottis and buccal cavity at the time of encounter. The snake and pentastomes were collected and stored at −20 °C. The snake was dissected and 61 adult *R. orientalis*, including 33 females (mean length = 55 mm) and 28 males (mean length = 12 mm), were removed from the mouth, trachea, lungs, and body cavity of the snake. All specimens were preserved in 70% ethanol following dissection.

Genomic DNA was extracted from a single adult female *R. orientalis* specimen using the Quick-DNA Miniprep kit (Zymo Research, Irvine, CA) according to the manufacturer’s guidelines. The entire pentastome was used (17.3 mg), thus was not archived, but the DNA is available under voucher ID Ror_cc_003 and additional specimens from the same host are archived at the University of Central Florida. A total of 1.4 µg of DNA was submitted to Novogene (Oxford, UK) for Illumina DNA sequencing. The genomic DNA was randomly fragmented, end-repaired, A-tailed, and ligated with Illumina adapters. The resulting fragments were PCR-amplified, size selected, and sequenced on a NovaSeq 6000 (Illumina, San Diego, CA) using paired-end 150-bp chemistry. A total of 354,419,038 paired reads were generated totaling 106.3 gigabases.

The raw reads were trimmed and filtered using *fastp v0.20.0* (Chen et al. 2018). Read trimming included adapter removal, trimming of both the 3′ and 5′ ends of bases with a Phred-scaled quality score (Q) <20, removal of 3′ bases if the 4-base mean *Q* < 20, and 3′ homopolymers of length ≥10. Entire reads were excluded if their length was <50, contained ≥5 or ≥30% uncalled bases, had a mean *Q* < 20, or if their complexity score was <30. The mitogenome assembly was created in two steps. First, a *de novo* assembly was generated using *mitofinder v1.4.1* (Allio et al. 2020) from 25% downsampling of the cleaned reads using *BBTools* (https://sourceforge.net/projects/bbmap/) and a reference composed of four existing pentastome mitogenomes: *Armillifer grandis* (NCBI accession NC_037187), *Armillifer agkistrodontis* (NC_032061), *Linguatula arctica* (NC_051998), and *Linguatula serrata* (NC_039399). Second, the largest mitochondrial scaffold (15,375 bp) from *mitofinder* was used as the seed for an assembly using *NOVOPlasty v4.3.1* (Dierckxsens et al. 2017) with a *k*-mer length of 39. *NOVOPlasty* assembled 1,357,433 read pairs to produce a final, circularized, mitogenome sequence 15,320 bp in length with approximately 29,000X coverage. The final sequence was deposited in NCBI (OP857516).

The resulting mitogenome was annotated with *MitoZ v3.4* (Meng et al. 2019) using the Invertebrate Mitochondrial Genetic Code (NCBI; transl_table = 5) and visualized using *OGDraw* v1.3.1 (Greiner et al. 2019). The mitogenome had a GC content of 33.1% and contained 13 protein-coding genes, two ribosomal RNA genes, and 22 tRNA genes (Figure 2). A tRNA-Cys was not identified by *MitoZ. S*ubsequent annotation using MITOS2 (Donath et al. 2019) identified a putative tRNA-Cys in the position consistent with Lavrov et al. (2004) (Figure 2), but this tRNA annotation was of low score and missing the characteristic D-loop and T-loop arms of tRNAs. Although mitochondrial tRNAs lacking structural arms are not uncommon, tRNA loss and rearrangements – even among closely related taxa – are themselves common (Salinas-Giegé et al. 2015). The order of mitochondrial protein-coding genes in *R. orientalis* was consistent with that described for Pancrustaceans (Boore 1999). The order of tRNA and rRNA genes included several rearrangements, which are a common feature of crustacean mitogenomes (Tan et al. 2019; Castellucci et al. 2022). Importantly, the *R. orientalis* mitogenome shares the position of tRNA-Leu2 (uaa) between *COX1* and *COX2* with other pentastome mitogenomes (Lavrov et al. 2004), which is considered the derived state in Pancrustaceans (Boore and Brown 1998; Boore 1999).

**Figure 2.**
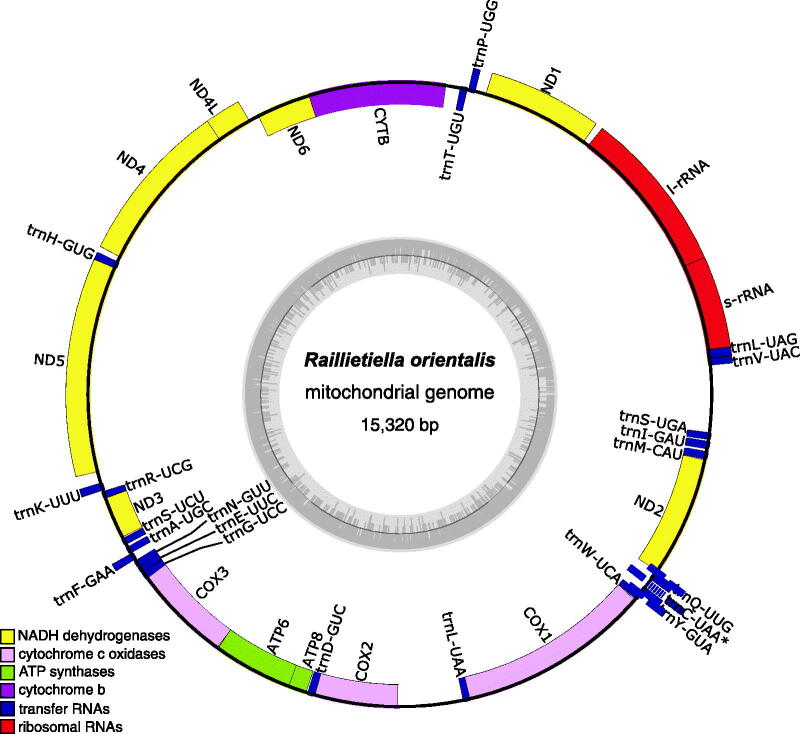
Map of the assembled and annotated *Raillietiella orientalis* mitochondrial genome (NCBI accession: OP857516). the 13 protein-coding genes, 22 tRNA genes, and two ribosomal RNA genes are shown as colored blocks according to the legend provided. The asterisk for trnC-UAA indicates that this tRNA annotation is incomplete. The grey inner circle represents the local GC content and the blue box highlights a putative at-rich, non-coding control region. The mitogenome map was created using *OGDraw* v1.3.1 (Greiner et al. 2019).

A phylomitogenomic analysis was performed using the *R. orientalis* mitogenome, 26 Pancrustacean mitogenomes (including the five pentastomes), and the Annelid outgroup *Lumbricus terrestris*. The thirteen protein-coding genes were prepared, retro-aligned, hyper-variable sites removed, and concatenated using *EZmito v2022.10* (Castresana 2000; Wernersson and Pedersen 2003; Cucini et al. 2021). A phylogenetic tree and 1000 ultrafast bootstrap replicates were constructed with IQ-TREE (Trifinopoulos et al. 2016) using an optimal partitioning strategy based on 39 partitions (i.e. protein-coding genes and codon position) and best-fit model for each partition selected by the lowest Bayesian information criterion value. Thirty-five partitions were fit with models TVM + F+I + G4 (ATP6/2 + CYTB/2, COX1/2, CYTB/1, NAD1/1, NAD2/1, NAD4/1, NAD4L/2, NAD6/2), GTR + F+I + G4 (ATP6/1 + COX3/1, COX2/1, COX3/2, NAD5/1, NAD/2), TVM + F+G4 (NAD1/2, NAD2/2, NAD4/2), TPM3u + F+ASC + G4 (COX1/3, COX2/3, CYTB/3), TPM2u + F+I + G4 (NAD4/3, NAD6/1), TIM3 + F + ASC + G4 (ATP6/3, NAD2/3), TPM2 + F + ASC + G4 (NAD1/3), TPM3 + F + ASC + G4 (COX3/3), TPM3u + F+G4 (NAD3/2), TPM2 + F + G4 (NAD4L/1), TPM2u + F+ASC + G4 (NAD4L/3), TIM2 + F + ASC + G4 (NAD5/3), SYM + I+G4 (COX1/1), SYM + G4 (NAD3/1), K3Pu + F+I (ATP8/1), GTR + F+G4 (COX2/2), F81 + F + I (ATP8/2), and HKY + F+ASC + G4 (ATP8/3 + NAD3/3 + NAD6/3). The resulting phylogenetic tree (Figure 3) clustered *R. orientalis* within a highly supported monophyletic clade containing the other pentastomes, and consistent with that generated using the 18S rDNA gene (Dajem et al. 2021). The tree also supported the relationship of the subclass Pentastomida within the Maxillopoda and sister to the subclass Branchiura.

**Figure 3.**
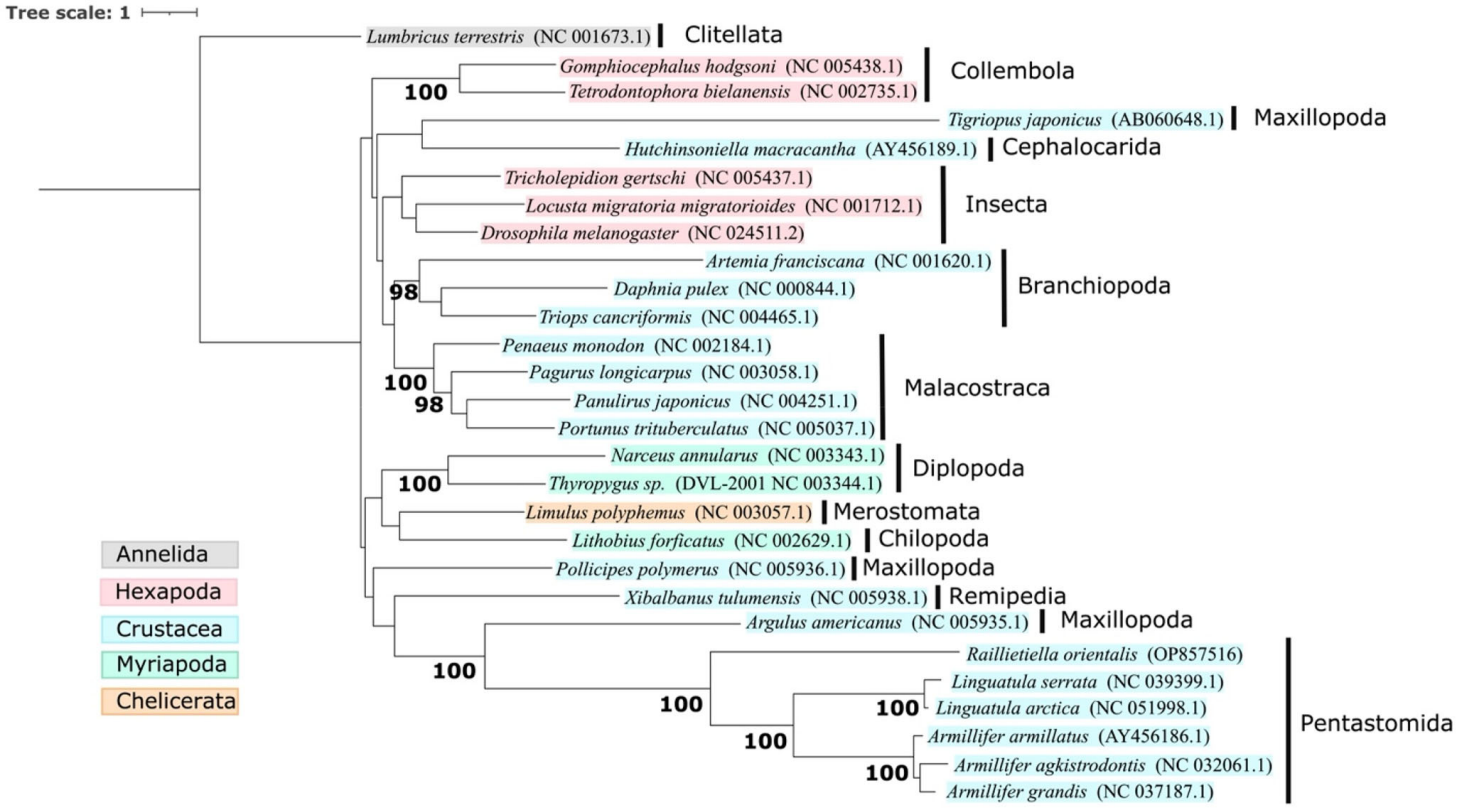
Maximum-likelihood phylogenetic tree based on 13 protein-coding genes from 27 Pancrustacean mitogenomes and 1000 ultrafast bootstrap replicates. Accession numbers are shown within parentheses, and the taxonomic classes are shown by vertical black bars. The Annelid *Lumbricus terrestris* was used as the outgroup. Nodes with ultrafast bootstrap support ≥95 are labeled.

## Data Availability

The mitogenome sequence assembly is available in NCBI (https://www.ncbi.nlm.nih.gov/) as accession OP857516. The associated BioProject, BioSample, and SRA accessions are PRJNA885072, SAMN31441700, and SRR22081628, respectively.
